# A Pilot Study to Evaluate Early Predictive Value of Thorax Perfusion-CT in Advanced NSCLC

**DOI:** 10.3390/cancers13215566

**Published:** 2021-11-06

**Authors:** Francisco Aya, Mariana Benegas, Nuria Viñolas, Roxana Reyes, Ivan Vollmer, Ainara Arcocha, Marcelo Sánchez, Noemi Reguart

**Affiliations:** 1 Department of Medical Oncology, Hospital Clínic, 08036 Barcelona, Spain; faya@clinic.cat (F.A.); nvinolas@clinic.cat (N.V.); rmreyes@clinic.cat (R.R.); aarcocha@clinic.cat (A.A.); 2 Translational Genomics and Targeted Therapies in Solid Tumors, IDIBAPS, 08036 Barcelona, Spain; 3 Centre for Genomic Regulation (CRG), The Barcelona Institute of Science and Technology, 08003 Barcelona, Spain; 4 Pompeu Fabra University, 08002 Barcelona, Spain; 5 Department of Radiology, Hospital Clínic, 08036 Barcelona, Spain; mnbenega@clinic.cat (M.B.); vollmer@clinic.cat (I.V.); msanche@clinic.cat (M.S.); 6 Thoracic Oncology Unit, Hospital Clínic, 08036 Barcelona, Spain

**Keywords:** imaging, perfusion CT, NSCLC, antiangiogenic, biomarkers, tumor perfusion, bevacizumab

## Abstract

**Simple Summary:**

The use of targeted drugs has brought about the development of new imaging techniques which are able to assess in vivo processes and changes in vascularization parameters can be captured as part of the antitumor response to antiangiogenic therapies. This pilot study (IMPACT trial, NCT02316327) aimed to explore the capacity of Perfusion-Computed Tomography (pCT) to detect early changes in tumor vascularization in non-small cell lung cancer (NSCLC) patients treated with an antiangiogenic-based therapy. Our results confirm the feasibility of pCT to capture early changes in tumor vasculature and suggest the potential of blood volume (BV) to early identify differential tumor responses to antiangiogenic therapy.

**Abstract:**

Background: The role of perfusion computed tomography (pCT) in detecting changes in tumor vascularization as part of a response to antiangiogenic therapy in non-small cell lung cancer (NSCLC) remains unclear. Methods: In this prospective pilot study (IMPACT trial, NCT02316327), we aimed to determine the ability of pCT to detect early changes in blood flow (BF), blood volume (BV), and permeability (PMB), and to explore whether these changes could predict the response at day +42 in patients with advanced, treatment-naive, non-squamous NSCLC treated with cisplatin and gemcitabine plus bevacizumab. Results: All of the perfusion parameters showed a consistent decrease during the course of treatment. The BV difference between baseline and early assessment was significant (*p* = 0.013), whereas all perfusion parameters showed significant differences between baseline and day +42 (*p* = 0.003, *p* = 0.049, and *p* = 0.002, respectively). Among the 16 patients evaluable for efficacy, a significant decline in BV at day +7 from baseline was observed in tumors with no response (*p* = 0.0418). Conclusions: Our results confirm that pCT can capture early changes in tumor vasculature. A substantial early decline of BV from baseline might identify tumors less likely responsive to antiangiogenic-drugs.

## 1. Introduction

In recent decades, lung cancer treatment has drastically changed, shifting towards personalized therapies with specific molecular therapies, including kinase inhibitors, antiangiogenics, and immunotherapies, which has led to a paradigm shift in the approach for lung cancer patients [1].

The role of the vascular endothelial growth factor (VEGF) in the stimulation of tumor angiogenesis, the maintenance of existing vessels, and the resistance to therapies, along with its negative prognostic significance in non-small cell lung cancer (NSCLC), have made it an important therapeutic target against this tumor [2]. Bevacizumab is a humanized anti-VEGF antibody that can function as an antiangiogenic link by binding directly to VEGF and deactivate it in the tumor microenvironment [3]. It was the first antiangiogenic drug approved for advanced lung cancer [4,5].

Traditionally, the evaluation of efficacy has been based purely on morphological data, by means of studying changes in the diameter of lesions according to the Response Evaluation Criteria in Solid Tumors (RECIST) [6]. However, evaluation of the response based exclusively on size change misses important biological and physiological information of the tumor that can be relevant in treatment decision-making [7]. To overcome this limitation, several functional imaging techniques, such as perfusion computed tomography (pCT), have been the subject of extensive research to evaluate the response to therapy in advanced NSCLC patients, particularly to antiangiogenic agents. However, the data is conflicting and most of these approaches have not been evaluated in clinical studies.

In this prospective pilot study, we aimed to assess whether pCT could capture early changes in the tumor vasculature of NSCLC patients treated with a combination of antiangiogenic therapy and chemotherapy. Secondly, we explored if perfusion parameters dynamics (early at day +7 and at day +42) correlate with tumor response to therapy (day +42).

## 2. Materials and Methods

### 2.1. Study Design and Patients

This is a single-arm, non-interventional, pilot study performed at a single institution. The study was done in accordance with the International Conference on Harmonization Good Clinical Practice guidelines, the Declaration of Helsinki, and applicable local regulations with approval from local ethics committees and institutional review boards. Written informed consent was obtained from all participants. This study is registered at ClinicalTrials.gov (NCT02316327) and has been completed.

Patients were recruited from July 2013 to April 2016 at the Hospital Clinic of Barcelona. Eligible patients had cytologically or histologically confirmed, advanced or metastatic non-squamous NSCLC (stage IV according to the seventh edition of the American Joint Committee on Cancer TNM staging system) for which they had not received prior systemic chemotherapy. All patients were required to have at least one unidimensional, measurable thoracic lesion of ≥1 cm as shown by conventional computed tomography (CT). Other eligibility criteria included a World Health Organization (WHO) performance status score of 0 or 1, suitability for first-line platinum-based chemotherapy, and an adequate organ and bone marrow function. Patients with brain metastases were eligible provided they were asymptomatic or treated and stable, and off steroids and anticonvulsants for at least one month before study entry.

The main exclusion criteria included a history of hemoptysis grade ≥2 (defined as 2.5 mL or more of fresh blood) within three months prior to treatment, mixed adenosquamous carcinoma, radiological evidence of compression or invasion of great blood vessels (i.e., pulmonary artery or superior vena cava), bleeding risk factors (such as coagulopathy, thrombolytic therapy within 10 days prior to treatment), and uncontrolled, concurrent illness or active infections. A complete description of all inclusion and exclusion criteria is included in Supplementary Materials. Adverse events were collected and graded according to the National Cancer Institute common terminology criteria for adverse events (NCI-CTCAE), version 4.0.

### 2.2. Treatment

Treatment was administered intravenously (IV) and consisted of chemotherapy with cisplatin at 80 mg/m^2^ administered on day 1 and gemcitabine at 1250 mg/m^2^ on days 1 and 8, plus Bevacizumab 7.5 mg/kg IV on day 1. Treatment was repeated every 21 days for up to six cycles. Patients with non-progressive disease were allowed to continue with bevacizumab monotherapy as maintenance until disease progression, unacceptable adverse events, withdrawal of consent, or death.

### 2.3. Imaging Protocol

Perfusion-CT was performed at day −1 (baseline), day +7 and day +42, followed by a CT of the thorax and abdomen to perform the RECIST 1.1 assessment. Then, only a CT of the thorax and abdomen was performed every two cycles until disease progression (Figure S1). A dual-source scanner with 128 detector rows (Flash Definition^®^, Siemens; Forchheim, Germany) was used.

An 18-gauge cannula was placed into a superficial vein of the antecubital fossa while the patient lay supine on the table. All patients were instructed to smoothly breathe during image acquisition to avoid excessive lung motion. No further preparation was necessary.

Fifty milliliters of iodinated contrast was injected (Iopromide 300, Ultravist^®^ Bayer Healthcare; Berlin, Germany) at 5 mL/s, followed by 50 mL of saline at the same rate. The pCT scan was initiated 5 s after the injection of the contrast commenced, using the following parameters: 80 kVp and 100 mAs; 0.33 s tube rotation time. The total time of the pCT study was always 45 s. The time interval between scans was 1.5 s. The total length of the studies along the z-axis was always 21 cm. After finishing the pCT, an additional dose of 50 mL of iodinated contrast was administered to perform the chest and abdomen CT.

The data was processed using a dedicated workstation (Multi-Modality Workplace^®^, Siemens, Forchheim, Germany) running the Syngo Volume Perfusion Computed Tomography (VPCT) Body program, VE36A. First, the automatic motion and noise correction algorithms included in the VPCT Body software were applied. An arterial density-to-time curve was obtained by placing a region of interest in the thoracic aorta. The tumor volume was selected via manual segmentation, drawing the contours of the lesion in the axial, coronal, and sagittal planes. For the perfusion evaluation, we included the best thoracic lesion to be segmented, including parenchymal lung tumors in 11 patients, mediastinal lymph nodes in 4 patients, and pleural metastases in 2 patients. The following perfusion parameters were calculated using a variant of the deconvolution algorithm: BF, in mL/100 mL/min; BV, in mL/100 mL; and PMB, in mL/100 mL/min. For the radiological response evaluation, the overall tumor burden was assessed according to RECIST v1.1 criteria [6]. Target and non-target lesions of non-thoracic lesions were also taken into account and the definitions of complete response (CR), partial response (PR), stable disease (SD), and progressive disease (PD) from RECIST 1.1 were used to categorize the overall response.

All perfusion parameters and efficacy assessments were performed by the same reader, a senior chest radiologist with broad experience in lung cancer and specific training in perfusion post-processing (M.S).

### 2.4. Statistical Analysis

The co-primary endpoints of the study were to assess early changes evaluated at day +7 after treatment in perfusion parameters (BF, BV, and PMB), and to correlate the perfusion parameters at different timepoints, as well as their changes with radiological tumor response, according to RECIST v1.1 (day +42).

This is a pilot study with an initial expected sample size of 20 patients. In order to decide the sample size of our study, we considered delving into the perfusion parameters of the responders’ population, and estimated that we had to include 20 patients to achieve the minimum of 3 responders, according to the formula given by Viechtbauer et al. (confidence 0.95, probability 0.15).

In the end, 19 patients signed the informed-consent form. The baseline and patient characteristics, as well as objective response rates were analyzed in the intent-to-treat (ITT) population, which included all patients who signed the informed consent. The per protocol (PP) population was all patients who received at least one cycle of treatment and were assessed with conventional and perfusion CT at day +7. Progression-free survival (PFS) and overall survival (OS) were analyzed in the PP assessed at day +42. The pCT parameters and characteristics, such as the type of thoracic target lesion, as well as overall survival and progression-free survival, were analyzed in the PP. PFS was defined as the time from treatment initiation until disease progression per RECIST v1.1 as assessed by the investigator or death from any cause. OS was defined as the time from treatment initiation until death from any cause.

We conducted univariate analyses using Fisher’s exact test for categorical factors and paired or independent Wilcoxon and Friedman tests for comparisons among continuous variables. The Bonferroni method was used for *p* value adjustments when needed for multiple comparisons. Survival curves were estimated using the Kaplan–Meier method and the log rank test was performed to compare PFS between groups. All statistical analyses were performed with R 4.0.3.

## 3. Results

### 3.1. Patient Characteristics

**Figure 1:**
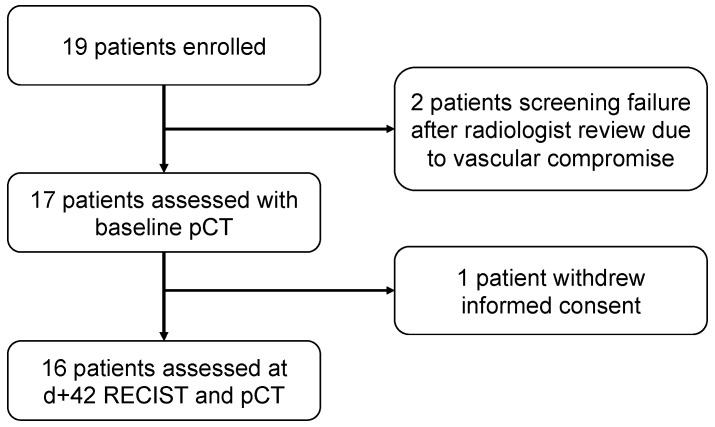
Study patients’ flow chart.

Between July 2013 and April 2016, 19 patients were enrolled. Of these, two patients did not receive treatment because of a protocol deviation due to vascular compromise (invasion or compression) and one patient withdrew consent after one cycle of treatment (Figure 1).

Fifteen patients were male, and the median age was 66 years old (range: 38–75 years old). All patients had a history of tobacco exposure and 10 patients were active smokers at screening. The most frequent histology was adenocarcinoma (17 patients, 89.5%), and 18 patients were diagnosed as stage IV (42.1% M1a and 52.6% M1b). No EGFR mutation nor ALK translocation were found, and 10 tumors (52.6%) harbored a KRAS mutation. Four patients were previously treated with surgery and two patients had received prior radiotherapy. The mean number of chemotherapy cycles was 4.5 (range 1–6) and the mean number of bevacizumab doses as maintenance was 7.5 (range 0–27). Twelve patients received at least one line of subsequent treatment (range 0–7), of which five patients were treated with immunotherapy. The median follow-up was 19.5 months (95% confidence interval [CI]: 10.8–31). Sixteen patients were suitable for response assessment at day +42. Among them, seven patients (36.8%) had a PR, eight (42.1%) had SD, and one patient (5.2%) showed PD as the best overall objective response, according to RECIST v1.1. The baseline characteristics are summarized in Table 1.

No unexpected adverse events were reported, and the safety profile was consistent with previously published data [4]. No deaths deemed by investigators to be related to the treatment were reported (Table S1).

### 3.2. Baseline Perfusion Parameters and Early Changes in Absolute Values

Baseline and early (day +7) assessments were performed of 17 patients (Figure 1) while day +42 assessment was performed of 16 patients. For the pCT evaluation and follow-up upon treatment, the selected baseline thoracic target lesions included lung nodules in 11 patients (64.7%), lymph nodes in 4 patients (23.5%), and pleura in 2 cases (11.8%).

**Figure 2:**
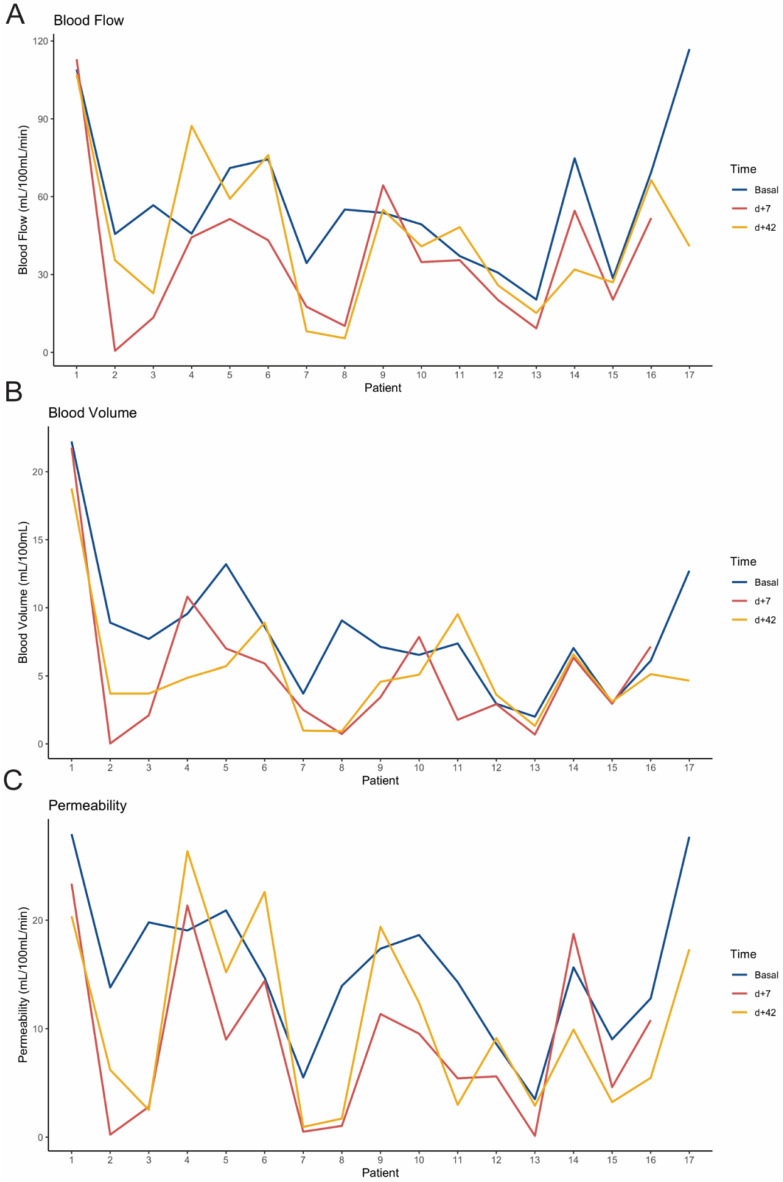
Line charts showing individual values of blood flow (**A**), blood volume (**B**), and permeability (**C**) at baseline (blue line), day +7 (red line), and day +42 (yellow line) after treatment initiation, case by case.

**Figure 3:**
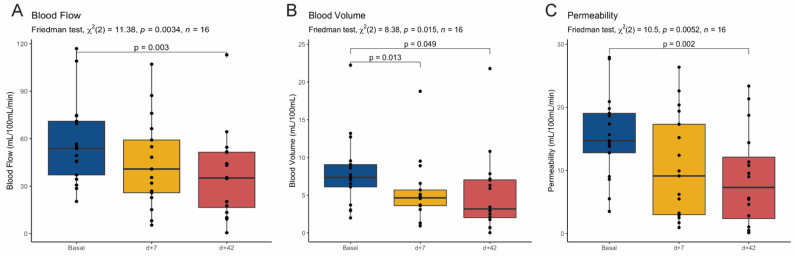
Boxplots showing values of blood flow (**A**), blood volume (**B**), and permeability (**C**) at baseline, day +7, and day +42 after treatment initiation in the entire population.

Individual measurements of perfusion parameters over time and over all patients are summarized in Figure 2. Mean values of the three perfusion parameters (BF, BV, and PMB) measured at baseline, early at day +7 and at day +42 showed a consistent decrease during the treatment (Figure 3). The mean values of the different perfusion parameters at the different time-points were significantly different among them (Table 2). Moreover, a paired Wilcoxon test between measurements at baseline and at day +42 showed statistically significant differences in BF (*p* = 0.003), BV (*p* = 0.049), and PMB (*p* = 0.002). Interestingly, a statistically significant difference between paired baseline and early measurement (day +7 after treatment) was also found for BV (*p* = 0.013) (Figure 3).

### 3.3. Percentage Change in Perfusion Parameters from Baseline

**Figure 4:**
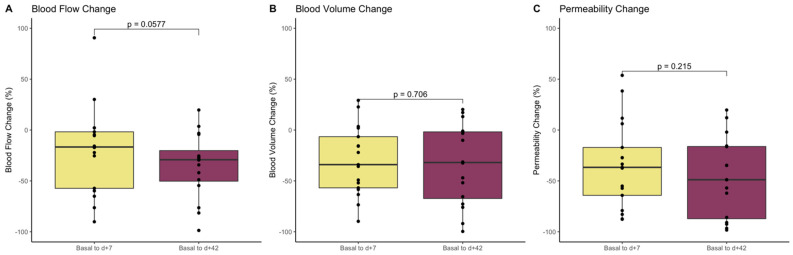
Boxplots showing percentage changes of blood flow (**A**), blood volume (**B**), and permeability (**C**) at day +7, and day +42 after treatment initiation compared to basal values in the entire population.

We then evaluated the percentage change of each perfusion parameter taking into account values from day +7 and day +42 in comparison to baseline values (Table 3 and Figure 4). No statistically significant differences were found between the early percentage change and the day +42 percentage change in any perfusion parameter.

### 3.4. Perfusion Parameters Changes and Tumor Response

The tumor response evaluation according to RECIST v1.1 and the perfusion parameters by pCT were evaluated at day +42 after treatment in 16 patients (Figure 1 and Table 4). Twelve patients (75%) were considered non-responders (including 11 patients with stable disease and 1 with progressive disease) and 4 patients (25%) were considered responders (all of them presented partial responses). Except for response rates, no differences were found in baseline and demographic data between responder and non-responder patients (Table 1).

**Figure 5:**
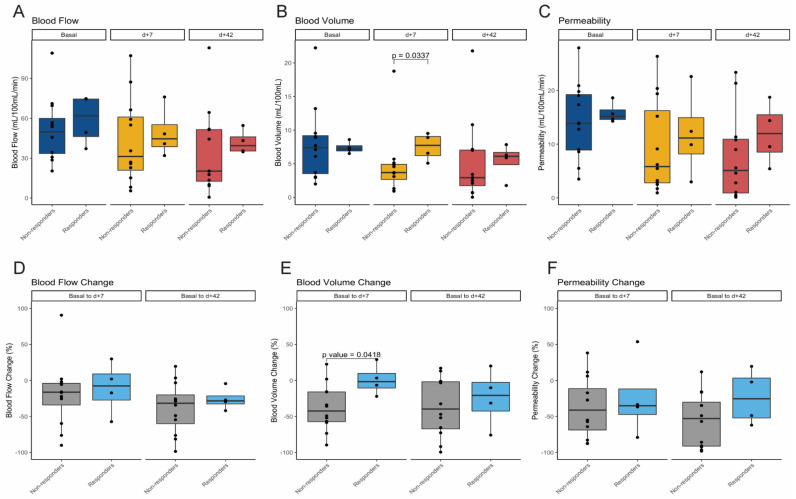
(**A**–**C**) Boxplots showing values of blood flow (**A**), blood volume (**B**), and permeability (**C**) at baseline, day +7, and day +42 after treatment initiation in non-responder and responder patients according to RECIST v1.1 at day +42. (**D**–**F**) Boxplots showing relative changes in percentages over time, at day +7, and at day +42 after treatment initiation in terms of blood flow (**D**), blood volume (**E**) and permeability (**F**) in non-responder and responder patients according to RECIST v1.1 at day +42.

**Figure 6:**
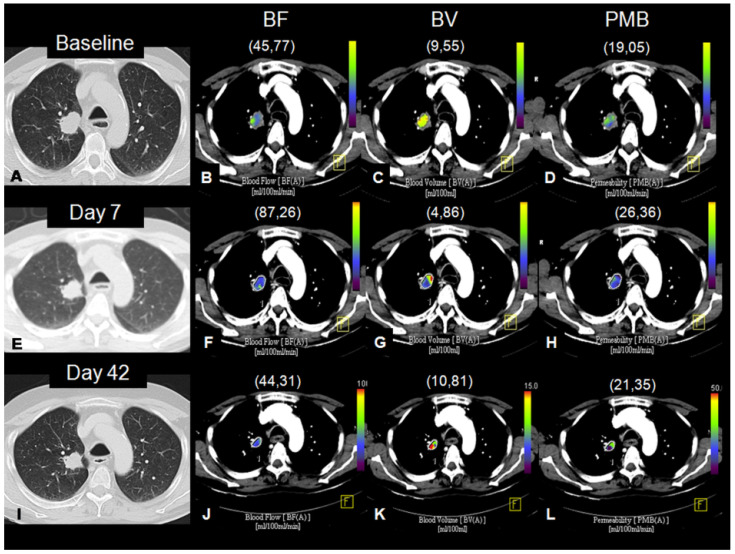
Axial thoracic CT of a 52-year-old man with stage IVa NSCLC showing a lung tumor in the right upper lobe, at baseline (**A**), day +7 (**E**), and day +42 (**I**). Perfusion CT of the lesion with parameters: Blood Flow, BF, (mL/100 mL/min) at baseline (**B**), day +7 (**F**), and day +42 (**J**); Blood Volume, BV, (mL/100 mL) at baseline (**C**), day +7 (**G**), and day +42 (**K**); Permeability, PMB, (mL/100 mL/min) at baseline (**D**), day +7 (**H**), and day +42 (**L**). Color-coded maps and quantification showed an early decrease in BV at day +7 with stable disease according to RECIST at day +42.

No statistically significant differences were found at baseline or at day +42 in terms of BF, BV, and PMB between responders and non-responders (Table 5). Early assessments at day +7 only showed significant differences in BV parameters, being lower in non-responders than responders (4.7 vs. 7.52 mL/100 mL, *p* = 0.0337) (Figure 5 and Figure 6). Consistent with the absolute values, a significant decline in the percentage of BV at day +7 from baseline was observed in non-responders with respect to responders (−38.01% vs. +1.02%, *p* = 0.0418) (Table 6 and Figure 5). No correlation was found between tumor burden in terms and BV neither at baseline nor at day +42 (Spearman’s rank correlation coefficient: −0.212 and −0.25, respectively).

### 3.5. Survival Analyses

The median PFS was 7.71 months (95% CI 5.48–14.92) and median OS was 17.6 months (95% CI 10.8–NA) with 14 deaths (87.5%) of the 16 patients undergoing follow-up.

We could not find significant differences in terms of PFS or OS in patients according to early BV changes, neither for other perfusion parameters at different time points.

## 4. Discussion

Our study was designed as a pilot study with the aim of deciphering the feasibility of pCT in a homogeneous cohort of advanced non-squamous NSCLC patients receiving cisplatin and gemcitabine plus bevacizumab as a first-line treatment. Our results meet both co-primary endpoints: first, indicating the capacity of pCT in capturing early changes at day +7 after treatment initiation; and, secondly, suggesting that a dynamic perfusion parameter, such as BV, might identify tumors less likely to respond to antiangiogenic therapy. To our knowledge, this is the first study carried out in a prospective manner showing the feasibility of pCT in detecting early changes as soon as day +7 after the first treatment administration in addition to at different time points.

The role of pCT imaging in tumor response assessment in lung cancer remains uncertain despite several publications attempting to elucidate it and, to date, no functional imaging technique has been routinely established for use in clinical practice. The incorporation of bevacizumab, the first antiangiogenic drug approved for the treatment of advanced NSCLC, raised high expectations regarding the usefulness of dynamic perfusion techniques to stratify outcomes in patients treated with antiangiogenic agents. However, the results in the literature are rather conflicting. This is most likely due to the notable variability in treatments (different chemotherapy combinations, targeted therapies, and/or radiotherapy), in the time points used for imaging acquisition, and in the criteria used for response evaluation, which is mostly limited to the same single lesion from which the perfusion parameters were measured [8,9,10,11,12,13,14,15] (Table S2).

Our results indicate the presence of early changes in tumor vasculature after therapy with an antiangiogenic agent that can be detected through BV measured with pCT. We also observed that the relative drop was not increased over time under treatment in any perfusion parameter, suggesting that an early evaluation at day +7 is indeed sufficient to capture significant tumor vasculature changes. Despite the small sample size, this study points BV as the most reliable and sensitive perfusion parameter and propose the limited capacity of BF and PMB in evaluating tumor response to antiangiogenics. This finding is consistent with other previous publications [13,14,15] which have seen a correlation between BV values and dynamics with response to therapy. Fraioli et al. [13] evaluated the role of pCT in NSCLC in a similar setting of patients treated with antiangiogenic therapy (bevacizumab) in combination with chemotherapy (carboplatin and paclitaxel). They also reported a significant decrease in terms of BF and PMB in the overall population and higher BV values in responders at different time points throughout the treatment. In the same line, Tacelli et al. [15], using newly defined concepts of BV and PMB (TTV, total tumor vascular volume, and TEF, total tumor extravascular flow, respectively), also found that both parameters decreased throughout the treatment, with higher values found among responders when chemotherapy was administered with bevacizumab.

On the contrary, in our pilot study, none of the perfusion parameters (BF, BV, and PMB) at baseline or at day +42 were predictive of response. These results contrast with other reports in which higher responses were seen in tumors with higher baseline BF and BV perfusion values [14]. Another striking observation of our pilot study is the identification of preliminary signals suggesting a potential role of BV as an early predictive biomarker as we observed a significant decrease and, consequently, a significantly lower BV at day +7, among the non-responder patient population. Some other studies have suggested a decrease in BV among responders in comparison to non-responders [8,9]. However, the decrease in BV in responders was not consistent and was only observed among patients treated with chemoradiation [8] and in those tumors of non-adenocarcinoma histology [9].

Even though our pilot study was exploratory in nature, it avoids some of the weaknesses of previous publications, such as the heterogeneity of lung cancer histologies, the flexible time points for imaging acquisitions and the wide variety of treatments in the same study, which might result in misleading conclusions. In our study, the outcomes are consistent with other pivotal trials in literature in the same first-line setting for NSCLC [4]. On the other hand, we believed that the use of cisplatin and gemcitabine as the chemotherapeutic backbone was optimal in order to prevent the confusing anti-angiogenic effect led by taxanes, which have been recognized as a strong modulator of angiogenesis [16,17,18]. This regimen allowed us to assess changes in the perfusion parameters mostly induced by the antiangiogenic bevacizumab. Unlike most previous publications, we applied RECIST v1.1 not only in the target perfusion lesion, but also in the whole patient for the assessment of the overall tumor burden. Evaluation of a single lesion may result in a misinterpretation of the response even if using RECIST criteria, and correlation of perfusion parameters with a complete evaluation of tumor response is a more realistic approach, mimicking a real clinical scenario. Moreover, both perfusion parameters and radiological assessments were conducted by the same observer, which gives a stronger internal validity, preventing inter-observer variability.

Along with this, we hypothesized that there can be two different biological scenarios that could explain the early drop of BV at day +7 in non-responder patients. First, the inability of BV (measured by pCT) to discriminate between differentiated and undifferentiated blood vessels. An increased density of pathological undifferentiated vessels (defined as intratumor CD34+ cells) has been described in tumors with high BV levels [19,20]. Interestingly, Zhao et al. [21] found a negative correlation between tumor shrinkage upon antiangiogenic therapy and the degree of vascular differentiation (CD31+/CD34– and CD31–/CD34+), suggesting that tumors bearing a higher proportion of undifferentiated vessels (CD31–/CD34+) display a greater susceptibility to bevacizumab. Therefore, it might be possible that those tumors with higher baseline BV values were enriched with undifferentiated vessels, thus having a greater initial drop of BV after antiangiogenic treatment, despite its poor response at day +42. Unfortunately, no data on the correlation between perfusion parameters and vascular differentiation has been reported so far. Secondly, although antiangiogenic therapy is hypothesized to revert tumor vasculature to a more normal state, therefore improving the drug delivery, several studies have reported a decrease in the delivery of chemotherapy due to the reduction in tumor perfusion induced by the antiangiogenic therapy [22,23]. Therefore, it could be feasible that the early drop of BV seen in tumors without response reflects a more undifferentiated vascular content in which chemotherapy delivery would be negatively affected, limiting tumor response. Correlative sequential histopathological analyses, as well as different antiangiogenic schedules within the context of a clinical trial, are warranted to elucidate our hypothesis.

This pilot study has inherent limitations as it was designed as exploratory to obtain an initial proof-of-concept on the potential of pCT parameters to capture early tumor changes. As, a pilot study we did not provide a meaningful sample estimate that might overcome the imprecision resulting of small study designs. Therefore, conclusions driven on the potential predictive value of pCT parameters must be taken with caution and considered only of descriptive nature. Secondly, we acknowledge that the use of an active comparator arm without bevacizumab could have provided more insights about the definitive causality of the antiangiogenic. We could have also increased the informative value of our results by using other radiomic parameters (that have been shown to be useful in building predictive models) [24,25], or using multiple target lesions. However, this would add further complexity for use in clinical practice.

Growing evidence suggests that VEGF inhibitors can modulate the tumor microenvironment by promoting the differentiation and function of immune cells, ultimately increasing the antitumor effect of immunotherapy [26]. With the advent of immunotherapy, novel combination approaches with antiangiogenic agents—such as lenvatinib (NCT03976375), ramucirumab (NCT03971474), sitravatinib (NCT03906071), or nintedanib (NCT02856425), among others—are currently under investigation in several phase II-III trials, particularly in lung cancer patients with acquired resistance to immunotherapy. Thus, the development of innovative imaging evaluation methods—especially for tumor response evaluation—still represents a largely unexplored area of study that warrants further investigation.

## 5. Conclusions

Our pilot study confirms the feasibility of pCT in detecting early perfusion changes in tumor vasculature of NSCLC treated with antiangiogenic therapy and suggests BV as the most reliable and sensitive measurement to identify tumors less likely responsive to antiangiogenic-drugs.

## Figures and Tables

**Table 1:** Demographic and baseline characteristics of patients.

Variation		*n* (%)	Non-Responders, *n* (%)	Responders, *n* (%)	*p* Value
**Sex**	Male	15 (78.9)	11 (91.7)	3 (75)	0.45
**Age**	median (range)	66 (38–75)	66.8 (41–74)	57.6 (38–75)	0.521
**Smoking Status**	Active smoker	10 (52.6)	5 (41.7)	3 (75)	0.569
	Former smoker	9 (47.4)	7 (58.3)	1 (25)	
**Histology**	Adenocarcinoma	17 (89.5)	12 (100)	4 (0)	1
	Non-adenocarcinoma	2 (10.5)	0	0	
**Stage**	IIIB	1 (5.3)	1 (25)	0	0.426
	IV-M1a	8 (42.1)	1 (25)	5 (41.7)	
	IV-M1b	10 (52.6)	2 (50)	7 (58.3)	
**KRAS Status**	Mutant	10 (52.6)	2 (50)	6 (50)	0.548
	Wild type	5 (26.3)	2 (50)	2 (16.7)	
	Unknown	4 (21.1)	0	4 (33.3)	
**Prior Treatment**	Surgery	4 (21.1)	2 (16.7)	0 (0)	0.728
	Radiotherapy	2 (10.5)	1 (8.33)	1 (25)	
	Chemotherapy	0 (0)	0	0	
**ECOG PS**	0	5 (26.3)	2 (16.7)	3 (75)	0.063
	1	14 (73.7)	10 (83.3)	1 (25)	
**Treatment, Mean (Range)**	CT + BVZ	4.5 (1–6)	4 (2–6)	5 (3–6)	0.494
	BVZ maintenance	7.5 (0–27)	4.5 (0–27)	9.5 (2–20)	0.36
**Overall Objective Response at Day +42**	PR	4 (21)	0	4 (100)	**<0.001**
	SD	11 (58)	11 (91.7)	0	
	PD	1 (5.2)	1 (8.33)	0	
	NE	3 (15.8)	-	-	
**Best Overall Objective Response**	PR	PR	7 (36.8)	3 (25)	**0.038**
	SD	SD	8 (42.1)	8 (66.7)	
	PD	PD	1 (5.2)	1 (8.33)	
	NE	NE	3 (15.8)	-	
**Subsequent Lines of Treatment**	0	5 (26.3)	5 (41.7)	0	0.299
	1	6 (31.6)	4 (33.3)	1 (25)	
	>1	6 (31.6)	3 (25)	3 (75)	

Abbreviations: *n*, number; ECOG PS, European Cooperative Oncology Group Performance Status; CT, chemotherapy; BVZ, bevacizumab; PR, partial response; SD, stable disease; PD, progressive disease; and NE, not evaluable.

**Table 2:** Perfusion parameters shown are mean values, with the standard deviation in parentheses.

Parameter	Baseline (*n* = 17)	Early Day +7 (*n* = 17)	Day +42 (*n* = 16)	*p* Value ^1^
Blood Flow (mL/100 mL/min)	57.23 (26.63)	44.27 (28.12)	36.52 (27.88)	**0.003**
Blood Volume (mL/100 mL)	8.11 (4.8)	5.36 (4.19)	5.25 (5.36)	**0.015**
Permeability (mL/100 mL/min)	15.48 (6.73)	10.51 (8.26)	8.68 (7.59)	**0.005**

^1^*p* value results from Friedman tests.

**Table 3:** Changes in perfusion parameters from baseline values. Mean values are shown, with the standard deviation in parentheses.

Parameter	Early Day +7 (*n* = 17)	Day +42 (*n* = 16)	*p* Value ^1^
Blood Flow (change from baseline, %)	−19.6 (43.1)	−34.9 (31.9)	0.058
Blood Volume (change from baseline, %)	−30.3 (34.0)	−33.4 (39.9)	0.706
Permeability (change from baseline, %)	−34.3 (42.6)	−47 (39.3)	0.215

^1^*p* value results from Wilcoxon tests.

**Table 4:** RECIST assessments in non-responder and responder patients. Mean values are shown, with the standard deviation in parentheses.

Parameter	Time	Non-Responders (*n* = 12)	Responders (*n* = 4)	*p* Value ^1^
Overall tumor burden of target lesions (mm)	Baseline	106 (79.1)	53.8 (22.1)	0.317
	Day 42	94.8 (74.6)	34.5 (14.4)	0.078
Percentage change (%)		−10.6 (12)	−36 (2.85)	**0.001**
Target lesion for perfusion evaluation (mm)	Baseline	39.2 (24.5)	34.8 (14.1)	0.856
	Day 42	34.5 (21.9)	21.5 (10.8)	0.362
Percentage change (%)		−12.3 (17.8)	−39.8 (7.56)	**0.013**

^1^*p* value results from Wilcoxon tests.

**Table 5:** Perfusion parameters in non-responder and responder patients. Mean values are shown, with the standard deviation in parentheses.

Parameter	Time	Non-Responders (*n* = 12)	Responders (*n* = 4)	*p* Value ^1^
Blood Flow (mL/100 mL/min)	Baseline	51.71 (24.02)	58.90 (18.79)	0.379
	Day 7	42.89 (32.24)	49.26 (19.03)	0.521
	Day 42	34.69 (31.97)	42.02 (9.19)	0.446
Blood Volume (mL/100 mL)	Baseline	7.96 (5.58)	7.38 (0.88)	0.953
	Day 7	4.70 (4.71)	7.52 (2.06)	**0.034**
	Day 42	5.17 (6.11)	5.46 (2.60)	0.599
Permeability (mL/100 mL/min)	Baseline	14.35 (7.06)	15.82 (1.96)	0.599
	Day 7	9.45 (8.66)	11.97 (8.13)	0.446
	Day 42	7.56 (8.00)	12.02 (5.79)	0.262

^1^*p* value results from Wilcoxon tests.

**Table 6:** Changes in perfusion parameters in non-responder and responder patients from baseline value. Mean values are shown, with the standard deviation in parentheses.

Parameter	Time	Non-Responders (*n* = 12)	Responders (*n* = 4)	*p* Value ^1^
Blood Flow (change from baseline, %)	Day 7	−18.78 (45.96)	−10.56 (36.69)	0.521
	Day 42	−38.01 (35.77)	−25.68 (15.69)	0.648
Blood Volume (change from baseline, %)	Day 7	−38.01 (32.08)	1.02 (21.51)	**0.042**
	Day 42	−36.41 (40.99)	−24.32 (40.43)	0.684
Permeability (change from baseline, %)	Day 7	−37.49 (41.77)	−23.89 (55.78)	0.77
	Day 42	−54.95 (37.73)	−23.31 (38.58)	0.212

^1^*p* value results from Wilcoxon tests.

## Data Availability

All of the data generated or analyzed during this study is included in this published article, and the supplementary information files are available from the authors upon reasonable request.

## Supplementary Materials

The following are available online at https://www.mdpi.com/article/10.3390/cancers13215566/s1, Supplementary Methods: Inclusion and Exclusion criteria; Table S1: Adverse events of any cause in the treated population; Table S2: Summary of main publications of perfusion CT assessments in lung cancer; Figure S1: IMPACT trial—study design. Parameters used for pCT evaluation: Blood Flow (BF, mL/100 mL/min), Blood Volume (BV, mL/100 mL), and Permeability (PMB, mL/100 mL/min).
