# Supplementary material for: A Pilot Study to Evaluate Early Predictive Value of Thorax Perfusion-CT in Advanced NSCLC
Source: Cancers (Basel). 2021 Nov 6;13(21):5566. doi: 10.3390/cancers13215566 (PMC8583202; doi:10.3390/cancers13215566)
Supplement: Supplementary file 1 [file cancers-13-05566-s001.zip › cancers-1351389-supplementary.pdf]

Supplementary Files

A Phase II/IV Study to Evaluate Early Predictive Value of Thorax Perfusion-Computed Tomography in Advanced NSCLC Patients.

Francisco Aya et al.

INDEX

SUPPLEMENTARY METHODS 2

SUPPLEMENTARY TABLES (S1, S2) 5

SUPPLEMENTARY FIGURES (S1) 6

SUPPLEMENTARY METHODS

INCLUSION AND EXCLUSION CRITERIA

INCLUSION CRITERIA

1. Written and signed informed consent prior to beginning study-specific procedures.
2. Histological or cytologically confirmed locally advanced or metastatic (IIIB/IV) non-squamous non–small cell lung cancer (NSCLC).
3. Ability to comply with the protocol.
4. Age ≥ 18 years.
5. Eastern Cooperative Oncology Group Performance status (PS) 0-1.
6. At least 1 unidimensionally measurable lesion ≥ 1 cm in lung according to RECIST (v.1.1), not previously irradiated.
7. Adequate hematological function: absolute neutrophil count (ANC) ≥ 1.5 x 10^9/L AND platelet count ≥ 100 x 10^9/L AND hemoglobin ≥ 9 g/dL (may be transfused to maintain or exceed this level).
8. Adequate liver function: Total bilirubin < 1.5 x upper limit of normal (ULN) AND aspartate aminotransferase (AST) AND alanine aminotransferase (ALT) < 2.5 x ULN in patients without liver or bone metastases; < 5 x ULN in patients with liver or bone metastases.
9. Adequate renal function: Serum creatinine ≤ 1.25 x ULN or calculated creatinine clearance ≥ 50 mL/min. Urine dipstick for proteinuria < 2+. Patients discovered to have ≥ 2+ proteinuria on dipstick urinalysis at baseline should undergo a 24-hour urine collection and must demonstrate < 1 g of protein in 24 hours.

10. International normalized ratio (INR) ≤ 1.5 and activated partial thromboplastin time (aPTT) ≤ 1.5 x ULN within 7 days prior to randomization, unless there is a prophylactic use of anticoagulants.
11. Patients with asymptomatic treated brain metastases are eligible for trial participation. Patients must complete treatment for brain metastases (radiotherapy with or without surgery, or stereotactic radiosurgery), including steroids, at least 28 days prior to randomization. Treatment with anticonvulsants at the time of randomization (i.e., ≥ 28 days) is allowed as long as the anti-convulsant is at a stable dose).
12. Patients must not be pregnant or breastfeeding. Patients of childbearing potential (defined as being neither > 2 years after last menstruation or surgically sterile) and whose partners are of reproductive potential must use a highly effective contraceptive method (permitted methods of birth control should have a failure rate of less than 1% per year and include implants, injectables, combined oral contraceptives, intrauterine devices [IUDs; only hormonal devices], sexual abstinence, or a vasectomized partner) during the trial and for a period of at least 6 months following the last administration of trial drug(s). Patients with an intact uterus (unless they have been amenorrheic for the last 24 months) must have a negative serum pregnancy test within 7 days prior to randomization into the trial.
13. Patients of reproductive potential must agree to use a highly effective contraceptive method (permitted methods of birth control, i.e. with a failure rate of less than 1% per year, include a partner using implants, injectables, combined oral contraceptives, IUDs [only hormonal devices], sexual abstinence, or prior vasectomy) during the trial and for a period of at least 6 months following the last administration of trial drug(s).

EXCLUSION CRITERIA

1. Prior systemic chemotherapy for advanced NSCLC.
2. Mixed, non–small cell and small-cell tumors or mixed adenosquamous carcinomas with a predominant squamous component.
3. History of hemoptysis ≥ grade 2 (defined as bright red blood of at least 2.5 mL) within 3 months prior to randomization.
4. Surgery (including open biopsy) or significant traumatic injury within 28 days prior to randomization, or anticipation of the need for major surgery during trial treatment.
5. Minor surgery, including insertion of an indwelling catheter, within 24 hours prior to the first bevacizumab infusion.
6. Evidence of tumor invading or abutting a major blood vessel (e.g., pulmonary artery or superior vena cava) on imaging.
7. Radiotherapy to any site for any reason within 28 days prior to randomization. Palliative radiotherapy to bone lesions within 14 days prior to randomization is allowed.
8. Current or recent (within 10 days prior to first dose of bevacizumab) use of aspirin (> 325 mg/day), clopidogrel (> 75 mg/day), or current or recent (within 10 days prior to first dose of bevacizumab) use of full-dose (i.e. therapeutic dose) oral or parenteral anticoagulants or thrombolytic agents for therapeutic purposes. Prophylactic use of anticoagulants is allowed.
9. History or evidence of inherited bleeding diathesis or coagulopathy with a risk of bleeding.
10. Active gastrointestinal bleeding.
11. Inadequately controlled hypertension (blood pressure: systolic >150 mmHg and/or diastolic >100 mmHg) within 28 days prior to randomization, or a history of hypertensive crises or hypertensive encephalopathy.
12. Clinically significant (i.e., active) cardiovascular disease (e.g. cerebrovascular accident [CVA] or myocardial infarction within 6 months prior to randomization, unstable

angina, congestive heart failure [CHF] New York Heart Association [NYHA] Class ≥ II, or serious cardiac arrhythmia), that is uncontrolled by medication or may interfere with administration of trial treatment.

13. Non-healing wound, active peptic ulcer, or untreated bone fracture.
14. History of abdominal fistula, gastrointestinal perforation, or intra- abdominal abscess within 6 months prior to randomization.
15. Known hypersensitivity to bevacizumab, cisplatin or gemcitabine or any of its excipients.
16. Known major hypersensitivity to iodinated contrast agents.
17. Any malignancy other than NSCLC within 5 years prior to randomization, except for adequately treated carcinoma in situ of the cervix, basal or squamous cell skin cancer, localized prostate cancer treated with curative intent, and ductal carcinoma in situ (DCIS) treated surgically with curative intent.
18. Evidence of any other disease, neurological or metabolic dysfunction, physical examination finding, or laboratory finding that gives reasonable suspicion of a disease or condition that contraindicates the use of an investigational or SOC drug used in this study or puts the patient at higher risk for treatment-related complications.

SUPPLEMENTARY TABLES

Table S1. Adverse Events of Any Cause in the Treated Population

	**Any Grade**	**Grade ≥ 3**
	**N (%)**	**N (%)**
Any Event	17 (100)	14 (82)
Event leading to death	1 (5.9)*	1 (5.9)*
Event leading to death that was attributed to treatment	0	0
Serious Adverse Event	8 (47.1)	8 (47.1)
Event leading to discontinuation of any treatment component		
Discontinuation of cisplatin	4 (23.5)	
Discontinuation of gemcitabine	3 (17.6)	
Discontinuation of bevacizumab	2 (11.8)	
Any adverse event†		
Asthenia	16 (94.1)	8 (47.1)
Neutropenia	12 (70.6)	8 (47.1)
Nausea	10 (58.8)	2 (11.8)
Anemia	10 (58.8)	2 (11.8)
Thrombocytopenia	9 (52.9)	6 (35.3)
Decreased appetite	9 (52.9)	1 (5.9)
Constipation	8 (47.1)	1 (5.9)
Dysgeusia	8 (47.1)	0

Arthralgia	7 (41.2)	0
Increased ALT	6 (35.3)	0
Diarrhea	6 (35.3)	2 (11.8)
Vomiting	6 (35.3)	0
Stomatitis	6 (35.3)	0
Chest Pain	5 (29.4)	0
Headache	5 (29.4)	0
Pyrexia	5 (29.4)	0
Dermatitis	4 (23.5)	1 (5.9)
Increased AST	4 (23.5)	0
Epistaxis	4 (23.5)	0
Abdominal Pain	4 (23.5)	0
Back Pain	4 (23.5)	0
Weigh Loss	4 (23.5)	0
Edema Limbs	3 (17.6)	0
Hiccups	3 (17.6)	0
Cough	3 (17.6)	0
Hoarseness	3 (17.6)	0
Dysphagia	1 (5.9)	1 (5.9)
Esophagitis	1 (5.9)	1 (5.9)
Febrile neutropenia	1 (5.9)	1 (5.9)
Hypocalcemia	1 (5.9)	1 (5.9)
Event of special interest related to bevacizumab (Any Grade)		
Hypertension	8 (47.1)	2 (11.8)
Epistaxis	4 (23.5)	0
Proteinuria	4 (23.5)	1 (5.9)
Hemoptysis	2 (11.8)	0
Thromboembolic event	2 (11.8)	1 (5.9)
Superficial thrombophlebitis	1 (5.9)	0

Abbreviations: N (%), number of patients (percent); ALT, alanine aminotransferase; AST, aspartate aminotransferase.

*One patient died due to progressive disease.

†Reported as adverse events of all grades occurring in at least 15% of patients or

any adverse events of grade 3 or 4 occurred. Common Terminology Criteria for Adverse Events (version 4.0) in all patients who received at least one dose of study drug.

Table S2. Summary of main publications of perfusion CT assessments in lung cancer

	Patient characteristics					Changes of perfusion			
**Study**	**Patients**	**Tumor type**	**Treatment**	**Time points of assessments**	**Criteria of response evaluation**	**BF**	**BV**	**PMB**	**Correlation of pCT and tumor response**
**Wang, 2009** (5)	35	ADC (n=18)<br>Squamous (n=17)	CT (54%)<br>RT (20%)<br>CT+RT (26%)[Ω]	- Baseline<br>- Follow-up (n=22)	Overall	ns	ns	ns	-Baseline: ↑BF.<br>-CT+RT (10 vs 3): higher ↓ in BV and BF
**Lind, 2010** (12)	23<br>(19 had BF measurements at all time points)	NSCLC	Erlotinib + sorafenib (NCT00722969)	-Pretreatment<br>- 3 weeks<br>- 6 weeks	Overall RECIST and Crabb	↓ (not measurable in 7 and 9 patients at week 3 and 6, respectively)[&]	NA	NA	-FUP1 and FUP2: ↓BF in responders and higher ↓BF.
**Fraioli, 2011** (8)	45	ADC	Carboplatin + paclitaxel + BVZ	- 9 days ± 3 before<br>- 42 days ± 4<br>- 89 days ± 2 (n=14)	Single lesion	↓	ns	↓	-Baseline: ↑BF and ↑PMB<br>-FUP2: ↑BF, ↑BV, ↓ TTP and ↑PMB
**Fraioli, 2013** (9)	50	ADC (n=22)<br>Squamous (n=14)<br>LCC (n=14)	CT + BVZ<br>CT[Ψ]	- 10 days ± 2 before (n=55)<br>- 91 days ± 3 (n=50)	Single lesion	↓	ns	↓	-Baseline: ↑BF and ↑BV in PR compared to PD
**Tacelli, 2013** (10)	40	ADC (n=35)<br>Squamous (n=3)<br>LCC (n=2)	CT + BVZ (42.5%)<br>CT (57.5%)	- Baseline<br>- 24.5 days ± 6.6	Single lesion and overall	NA	↓TTV only in BVZ group†	↓TEF only in BVZ group†	-FUP1: higher ↓TTV in responders with BVZ<br>-FUP2: higher ↓TTV in responders with BVZ.

				- 62.9 days ± 9.9 (n=34)<br>- 159.9 days ± 50.9 (n=26)					
**Sudarski, 2015** (7)	100	ADC (n=56)<br>Squamous (n=28)<br>SCLC (n=16)	CT[‡]	-Pretreatment<br>- After 2 cycles of CT (median span between times: 44 days)	Single lesion	ns	ns	↓	No significant differences were found in BV, BF or PMB.
**Yabuuchi, 2018** (11)	66	ADC (n=57)<br>Squamous (n=7)<br>Large cell neuroendocrine (n=2)	BVZ + CT (30%)<br>Platinum-based CT (38%)<br>Other (32%)[#]	- Baseline<br>- 6-8 weeks after 2 courses	Single lesion. RECIST not used	Baseline bronchial artery perfusion (BAP) correlated with tumor reduction after 2 courses of CT + BVZ			
**Trinidad-López, 2019** (6)	53	ADC (n=28)<br>Squamous (n=16)<br>Undifferentiated (n=5)<br>LCC (n=3)<br>Neuroendocrine (n=1)	CT with platinum (86.8%)<br>CT + RT (13.2%)	- Baseline (29.4 ± 15.8 days before CT start)<br>- 63.9 days ± 28.1 after CT	Single lesion	ns	↓	ns	-Baseline: no differences.<br>-FUP1: all PR had a higher ↓BV. The decrease was not significant in the ADC only group.

Abbreviations: BF, blood flow; BV, blood volume; PMB, permeability; pCT, perfusion computed tomography; ADC, adenocarcinoma; LCC, large cell carcinoma; BV, bevacizumab; CT, chemotherapy; ns, no significant changes; NA, not assessed; NR, not reported; RECIST, Response Evaluation Criteria in Solid Tumors

[Ω] First-line platinum-based chemotherapy. Radiotherapy consisted of 60 Gy in 30 fractions of 2 Gy each.

[&]Not evaluable patients were assigned a BF value of zero.

[$]According to Crabb criteria: PR in 11 patients, SD in 10 patients.

[Y]Non-squamous carcinoma regimens: carboplatin + paclitaxel + bevacizumab or cisplatin + gemcitabine + bevacizumab; squamous carcinoma regimens: cisplatin, docetaxel, cisplatin + vinorelbine or cisplatin + gemcitabine.
[†]Newly defined parameters were calculated. Total tumor vascular volume (TVV, ml) was calculated as TTV = BV x VPCT, being VPCT the total volume of voxels included in the analysis and total tumor extravascular flow (TEF, ml/min) as TEF = Ktrans x VPCT, being Ktrans the volume transfer constant also called "flow extraction product" (ml/100 ml/min).
[‡]SCLC patients received cisplatin/carboplatin + etoposide, etoposide monotherapy or irinotecan + cisplatin. Non-small cell lung cancer patients received cisplatin/oxaliplatin/carboplatin + gemcitabine/vinorelbine, carboplatin/cisplatin + paclitaxel, gemcitabine monotherapy, vinorelbine or docetaxel.
[#]Carboplatin + pemetrexed + bevacizumab (n=20), Carboplatin + pemetrexed (n=13), EGFR inhibitor (n=11), Carboplatin + paclitaxel (n=8)
Docetaxel (n=5), Pemetrexed (n=3), Crizotinib (n=2), Cisplatin + pemetrexed (n=2), Cisplatin + vinorelbine (n=1), Cisplatin + gemcitabine (n=1)

SUPPLEMENTARY FIGURES

Figure S1. IMPACT trial – study design.

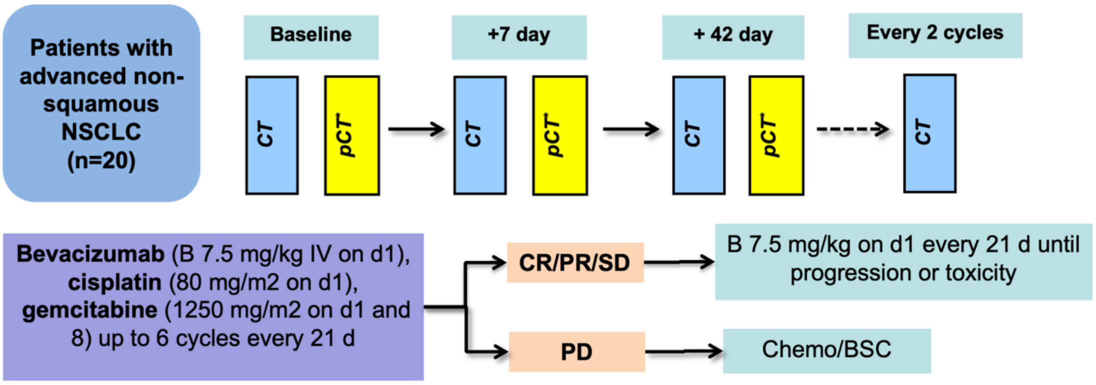

Parameters used for pCT evaluation: Blood Flow (BF, mL/100 mL/min), Blood Volume (BV, mL/100 mL), and Permeability (PMB, mL/100 mL/min). NSCLC, non–small cell lung cancer; CT, computed tomography; pCT, perfusion computed tomography; IV, intravenous; CR, complete response; PR, partial response; SD, stable disease; PD, progressive disease.
